# Principles of Forensic Medicine

**Published:** 1845-05

**Authors:** 


*Principles of Forensic Medicine.* By WILLIAM A. GUY, M. B. Cantab; Fellow of the Royal College of Physicians; Professor of Forensic Medicine King's College, London, &c. &c. First American Edition. With Notes and Additions, by CHARLES A. LEE, M. D. Professor of Materia Medica and General Pathology in Geneva College, &c. &c. 8vo. pp. 711. New York, 1845.

The appearance of two works, like those of Mr. Taylor and Dr. Guy, about the same period, and in a country—Great Britain—where the valuable Treatise of our friends and countrymen—the Doctors Beck—had reigned so long predominant, would seem to indicate a greater degree of attention to the subject of Forensic Medicine than has hitherto prevailed. An impulse has perhaps been given to their production by several interesting questions of a medico-legal nature, which have, of late years more especially, agitated our own as well as a sister profession.

Of the American edition of Mr. Taylor's work we have already spoken in terms of commendation; and likewise of the English edition of Dr. Guy's "Principles." Our attention will, therefore, be mainly directed to the additions which have been made by the American Editor. Before, however, we proceed to this examination, we may express our regret, that so much acerbity has been indulged in by Messrs. Taylor and Guy in regard to disputed questions of *originality*,—a word which, by the way, we are heartily tired of hearing in the sense in which it is usually employed. Dr. Guy was a pupil of Mr. Taylor,—followed his lectures at Guy's Hospital, and necessarily imbibed the tone of reflection, and often, doubtless, the very words that were employed by the master. It would have been difficult, indeed, for him to avoid this, and scarcely desirable if it had been practicable,—provided the tone and words of Mr. Taylor had been worthy of imitation, and we have no doubt they were.

It was but necessary that Dr. Guy should express his acknowledgments to Mr. Taylor, which he has done in a general manner in his Preface, so far as regards a published work of the latter, but this partial acknowledgment does not appear to have been entirely satisfactory. We think that Mr. Taylor ought to have been content with this reference to one of his works; although it might have been more creditable to Dr. Guy if he had likewise referred to his indebtedness to the lectures of Mr. Taylor, provided his notes of those lectures had been freely—as is affirmed—employed by him. When we see—as we have seen—even in the pages of one of our prominent medical periodicals, whole passages taken from the works of another, and copied as *editorials*, with scarcely the change of a word or letter, and without the mention even of the name of the real author, we are gratified to find even a general acknowledgment to a work that has been extensively used by an author, inasmuch as such acknowledgment indicates no attempt at concealment; or at all events points out to one, who is desirous to investigate the truth, the real source. Unquestionably, where facts, opinions, and views peculiar to a writer, or presented by him in a novel and striking manner, are cited, full credit ought to be given to the original observer; but it would be idle to load the pages of scientific treatises with such references, in the case of common descriptions,—as of ordinary surgical operations, articles of the materia medica, &c., in which there can necessarily be but little more difference of expression than would exist in describing a common geometrical figure. Leaving this disputed question, however, we may reiterate our conviction, expressed on a former occasion, that Dr. Guy's work is one of great value; and after a careful examination of both it and the "Manual" of Mr. Taylor, we are equally convinced, that the reflecting practitioner may advantageously possess himself of both one and the other. When reprints of works like this of Dr. Guy, or the Anatomy of Cruveilhier, make their appearance, we hail their advent with real pleasure, and frankly state our opinion of the exalted merits of the originals; whilst we as frankly animadvert on those of an inferior grade of merit,—such as that of Chailly on Midwifery, or of Magendie on Physiology,—and we instance these works—admitted by competent observers to be imperfect exponents of the state of Obstetrics and Physiology of the day—because it has been unworthily suggested by interested parties, that motives of a sectional character, which we contemn, have unduly biassed us in our judgment.

In his preface, after a somewhat overstrained eulogy of the work of Dr. Guy, the American Editor thus expresses himself.

"To the Editor was assigned the task of revising the text, correcting errors, and adapting the publication to the existing laws and institutions of this country. Leaving the text in its integrity, he has, accordingly, taken the liberty of making somewhat extensive additions, (in brackets,) citing numerous American authorities and decisions, with ample references, to render the work in the highest degree useful to those classes for which it is especially designed. * * * * The Editor has freely used the materials within his reach, giving due credit, however, to the sources whence his information has been derived. The recent Manual of Mr. Alfred Taylor has furnished some very valuable details, which will be found under their appropriate subjects." p. vi.

In casting our eyes over the book, we find the additions numerous, and, in most cases, judicious and pertinent. Dr. Lee is not, however, as rich in home authorities as we had expected from his well known industry. For example, under the head of Hypospadias as a cause of impotence, we find one additional case only cited by him; and that occurring to his respectable townsman Dr. Francis,—who, by the by, appears to be the great medical authority with the writers of the "Commercial Emporium"—whilst had he referred to the works on Medical Jurisprudence and Physiology at his elbow, he might have culled many equally worthy of being cited—not that it was important, however, to adduce either them or that of Dr. Francis. We confess, too, that we scarcely expected to see in a medical work the case of "Tittlebat Titmouse, Esq.," brought forward as a part of medical history!

In an addition, at page 57, Dr. Lee adopts the common opinion, that the long continued and excessive use of iodine has occasioned absorption of the testis, citing a case that had occurred in

New York. We do not deny the possibility of such absorption. We know that it has been asserted over and over again. Yet we must say, that after an observation of cases without number, in which the various preparations of iodine have been pushed as far as the system would bear them, we have not met with a solitary example, nor have we heard of one in the practice of any one of our friends.

Under the "signs of pregnancy" we find no reference to the results of experiments made in this country on Kiesteine by Drs. Perry and McPheeters, and by Dr. Kane; the last of whom has published the most satisfactory monograph on Kiesteine that we possess.

On the subject, too, of the uterine murmur as a sign of pregnancy (p. 98) it would have been advisable to state, that there is much reason for, the belief that it indicates merely the presence of a solid body pressing on the great vessels of the mother; and, therefore, is by no means pathognomonic of pregnancy. So, also, in regard to the corpus luteum, it would have been well to refer to the views of Négrier, Raciborski, Bischoff, and others, as respects the regular discharge of infecund ova at each menstrual period, and to the bearing which these views have on the question of corpora lutea as evidences of conception.

But, we admit that it is easy to suggest omissions; and to overlook the valuable additions that have been made. On the whole, we do not hesitate to say, the American Editor has most industriously and, we think, ably executed his task; and his labours are deserving of the patronage of his professional brethren.

